# Nod2: A Critical Regulator of Ileal Microbiota and Crohn’s Disease

**DOI:** 10.3389/fimmu.2016.00367

**Published:** 2016-09-20

**Authors:** Tabasum Sidiq, Sayuri Yoshihama, Isaac Downs, Koichi S. Kobayashi

**Affiliations:** ^1^Department of Microbial Pathogenesis and Immunology, College of Medicine, Texas A&M University, College Station, TX, USA

**Keywords:** NOD2, ileal Crohn’s disease, Paneth cells, microbiota

## Abstract

The human intestinal tract harbors large bacterial community consisting of commensal, symbiotic, and pathogenic strains, which are constantly interacting with the intestinal immune system. This interaction elicits a non-pathological basal level of immune responses and contributes to shaping both the intestinal immune system and bacterial community. Recent studies on human microbiota are revealing the critical role of intestinal bacterial community in the pathogenesis of both systemic and intestinal diseases, including Crohn’s disease (CD). NOD2 plays a key role in the regulation of microbiota in the small intestine. *NOD2* is highly expressed in ileal Paneth cells that provide critical mechanism for the regulation of ileal microbiota through the secretion of anti-bacterial compounds. Genome mapping of CD patients revealed that loss of function mutations in *NOD2* are associated with ileal CD. Genome-wide association studies further demonstrated that *NOD2* is one of the most critical genetic factor linked to ileal CD. The bacterial community in the ileum is indeed dysregulated in *Nod2*-deficient mice. *Nod2*-deficient ileal epithelia exhibit impaired ability of killing bacteria. Thus, altered interactions between ileal microbiota and mucosal immunity through *NOD2* mutations play significant roles in the disease susceptibility and pathogenesis in CD patients, thereby depicting NOD2 as a critical regulator of ileal microbiota and CD.

## NOD2, A Member of the NLR Family, Regulates Innate and Adaptive Immune Responses

NLR [Nucleotide binding domain (NBD)-leucine rich repeats (LRR) containing or nucleotide-binding oligomerization domain (NOD)-like receptor] proteins play an important role in shaping the immune system. Humans have 22 NLR protein members and mice have 34 members whereby abundance may vary in relation to cell types (1–4). While certain NLR protein members are critical for the early embryogenesis (5, 6) or upregulation of major histocompatibility complex (MHC) molecules (4, 7–10), other group of NLR proteins have important roles in recognizing pathogen and damage-associated molecular patterns (PAMPs and DAMPs, respectively) in the cytoplasm and eliciting innate immune responses (1, 11–16). NLRs have a tripartite structure consisting of an amino-terminal effector domain, a central NBD, and a carboxy-terminal LRR (17). Most NLR proteins contain distinct protein–protein association domains, such as caspase recruitment domain (CARD), pyrin domain (PYD), or a baculovirus inhibitor repeat (BIR). These variations of the amino-terminal domain are used to categorize NLRs into major subfamilies, which include NLRCs (NLRs containing CARD), NLRPs (NLRs containing Pyrin), and others (1, 3, 11, 14, 18–20). The central NBD, found in all NLRs, is involved in oligomerization and activation (1, 3). The carboxy-terminal LRRs may be used for the recognition of PAMPs and DAMPs by many NLRs, except NLRP4 and NAIP (Neuronal Inhibitor Apoptosis Protein) that do not use LRRs for ligand recognition (1, 3, 20–24).

NOD2 is a 110 kDa cytosolic protein (1040 amino acids) with two CARD domains, thereby is a part of the NLRC subfamily (13, 15). Known as a major genetic risk factor for Crohn’s disease (CD), *NOD2* gene is located on human chromosome 16p21. Upstream of the transcription start site of *NOD2*, are two NF-κB-binding sites within the promoter at 26 and 301 bp as well as vitamin D receptor-binding sites within the *NOD2* gene (25–27). *NOD2* is highly expressed in myeloid cells, such as dendritic cells and macrophages, but expression is low in T cells (27–30). Furthermore, *NOD2* is highly expressed in Paneth cells in the ileum with a lesser degree in epithelial cells from the intestines, oral cavity, and lungs (28, 29, 31–33). *NOD2* expression is induced by bacterial components (e.g., LPS), short-chain fatty acids (e.g., butyrate), hormonal vitamin D (1,25-dihydroxyvitamin D_3_), and pro-inflammatory cytokines (e.g., TNF-α) (25, 27, 28, 34–39). The expression of *NOD2* and downstream kinase, receptor-interacting serine/threonine-protein kinase 2 (*RIP2)* in the intestine is largely dependent on the presence of gut microbiota as the expression of these two genes is significantly low in germ-free mice and can be restored by colonization of single strains of bacteria or microflora from mice under SPF conditions (28, 31, 35).

It has been shown that NOD2 recognizes muramyl dipeptide (MDP), *N*-acetylmuramyl-l-alanyl-d-isoglutamine (40–42). LRRs at C-terminus are responsible for direct recognition of MDP, although affinity is not strong for *in vitro* association assays (13, 15). MDP is derived from peptidoglycan found mainly in Gram-positive bacteria and to a lesser extent in Gram-negative bacteria. MDP can be transported into the cytoplasm of mammalian cells from endo/phagosome through transporters, such as solute carrier family protein 5 (SLC15A4) (43–45). Both MDP recognition and NOD2 oligomerization are enhanced when ATP binds to the Walker A motif in the NBD, known to regulate the activity of most NLRs (13, 15).

**Figure 1:**
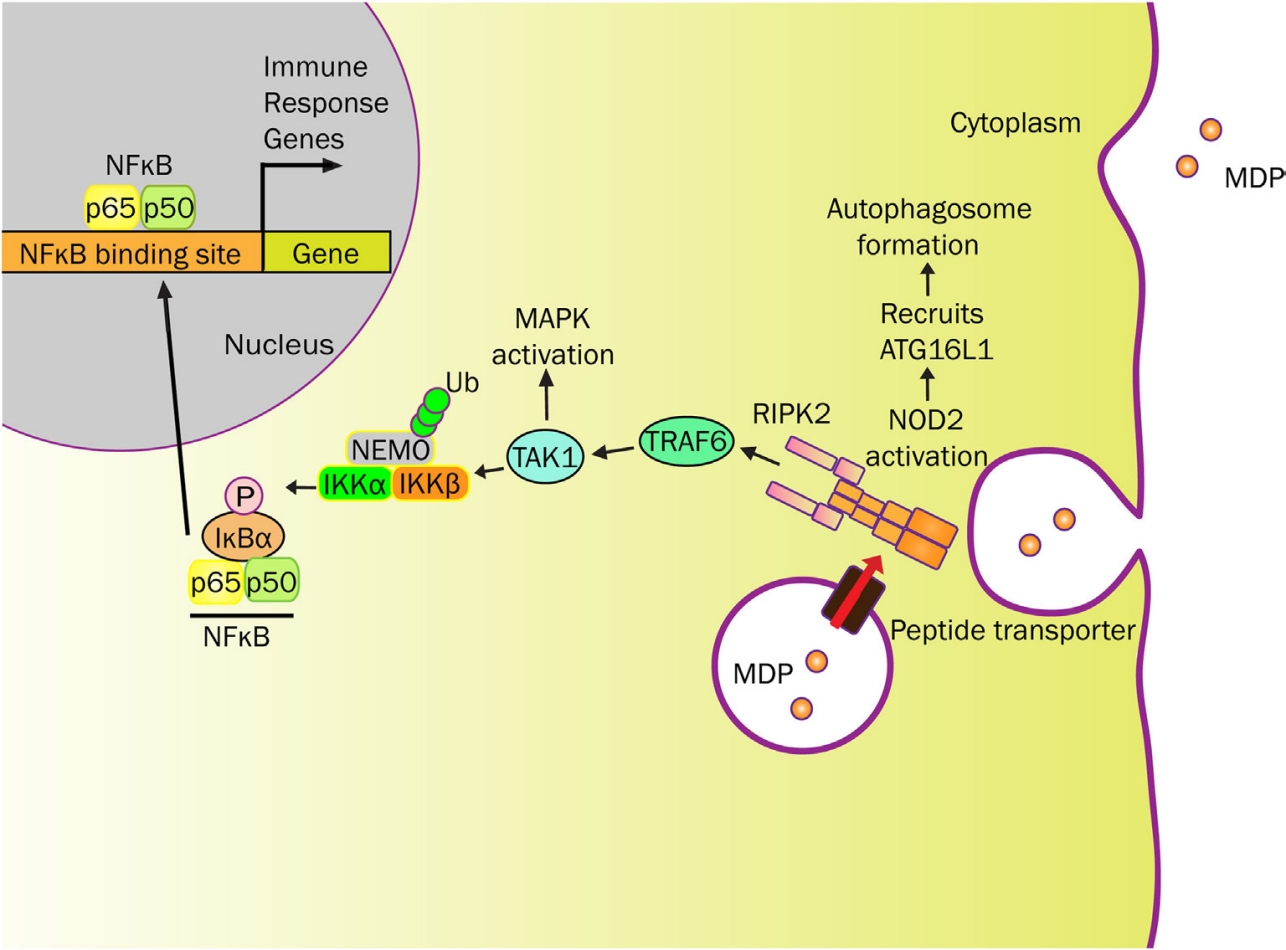
**NOD2 senses MDP and elicits immune responses**. MDP-derived from bacterial cell wall can be transported into the cytosol by endo/phagosome through peptide transporters (e.g., SLC15A4). MDP activates NOD2 to initiate RIPK2 recruitment resulting in platform formation. This cytosolic NOD2-initiated structure localizes E3 ligases (e.g., TRAF6) to ubiquitinate NEMO, for which NEMO and TAK1 forms a complex near IKK. In addition to activating the mitogen-activated protein kinase (MAPK) pathway, TAK1 phosphorylates IKK to initiate the NF-κB signaling pathway. IκB-α is phosphorylated by IKK, which targets IκB-α to proteasome degradation. No longer restricted, free NF-κB translocates into the nucleus and activates various immune response genes. NOD2 also recruits ATG16L1 near the plasma membrane to promote autophagosome formation, known to degrade antigenic structures. The release of cytokines and chemokines, initiated by NOD2, recruits immune cells to influence Th1 and Th2 responses.

NOD2 activation promotes oligomerization and recruitment of RIP2 by CARD-CARD homophilic interactions (Figure 1) (36, 46–48). E3 ligases [TNF receptor associated factor 6 (TRAF6), TNF receptor associated factor 2 (TRAF2), cellular inhibitor of apoptosis protein (cIAP2)] are recruited to this NOD2-RIP2 platform to ubiquitinate NEMO (NF-κB essential modulator) at amino acid R285 and R399 (42, 47, 49, 50). Whereas NOD2 is subsequently ubiquitinated and undergoes proteasomal degradation (51), TGFβ-activated kinase 1 (TAK1) and ubiquitinated NEMO form a complex within proximity to activate IκB kinase (IKK) that initiates the NF-κB signaling pathway (37, 46, 50). Once phosphorylated by IKK, IκB-α is targeted to proteasomal degradation that permits free NF-κB to translocate into the nucleus (35, 42, 46, 47, 50) (Figure 1).

In addition to NF-κB activation, the mitogen-activated protein kinases (MAPK), such as p38 and ERK, are activated in a TRAF6-dependent manner (42, 50) (Figure 1). Activation of these signaling cascades upon NOD2 stimulation induces the production of a variety of cytokines, chemokines, and antimicrobial peptides (TNF-α, IL-6, IFN-γ, IL-1β, IL-10, IL-8/CXCL8, α-defensin) depending on cell types (30, 52). NOD2 signal cascade is enhanced by toll-like receptor (TLR) agonists through NF-κB. Along with MDP, this relationship further highlights the impact of microbial components on the NOD2 pathway (1, 30, 53).

Through the innate immune system, NOD2 provides a defensive strategy to protect the hosts against bacterial infection. Various epithelial cells throughout the human body not only create a physical barrier but also produce antimicrobial peptides to regulate bacterial colonization (29, 31, 54). In addition to *NOD2* gene, mutations in *ATG16L1* (Autophagy-related 16 Like 1) gene are a risk factor for CD (55). ATG16L1 is a component of a large protein complex essential for autophagy, a mechanism that degrades intracellular components using the lysosome. As NOD2 is capable of recruiting ATG16L1 to the plasma membrane at the bacterial entry site, *NOD2* mutants failed to recruit ATG16L1, ultimately impairing autophagosomal encapsulation of invading bacteria in dendritic cells (56, 57). Therefore, both ATGL16 and NOD2 have interrelated roles for regulating the microbial invasion that has yet to be studied in Paneth cells (Figure 1). However, in Paneth cells, NOD2 recruitment onto dense vesicles helps coordinate cargo-sorting events that support host defenses (58). Therefore, NOD2 function and related responses regulate interactions between innate immunity and the intestinal microbiota.

As noted above, NOD2 and TLR signaling cooperate to enhance immune responses. This synergy of NOD2 and TLR activation affects the polarization of T cell adaptive immunity (Th1 vs. Th2 immune responses) (42, 52, 59, 60). While it is well documented that the NOD2 pathway cooperates with signals through NOD1 or various TLRs (e.g., TLR3, TLR4, TLR9) to enhance Th1 immune responses, Th2 responses are also induced through the cooperation of NOD2 with TLR2 agonists (35, 52, 61, 62). Regarding humoral immunity, NOD2 is capable of influencing antigen-specific immunoglobulin production based on studies using *Nod2*-deficient mice (42, 52). Thus, *NOD2* expression impacts immune responses as well as bacterial killing and colonization in the ileum (25, 31, 34, 35, 52, 61–63). In addition to bacterial components, dietary fiber as well as vitamin D-containing products also induces expression of *NOD2* to further increase the regulation of the microbiota (26, 28, 35).

## Etiology and Pathophysiology of Crohn’s Disease

Crohn’s disease is a chronic, relapsing inflammatory disorder of the gastrointestinal tract, most commonly involving the ileum and colon. CD patients typically suffer from frequent and chronically relapsing flares with diarrhea, abdominal pain, rectal bleeding, and malnutrition. Histologically, CD is characterized by transmural infiltration of lymphocytes and macrophages with granuloma. Because of the clinical and histological features, most CD patients require lifelong treatment, such as medication and surgery. For efficient treatment of CD, it is important to identify patients who carry risk factors for complications of the disease and initiate appropriate therapy at early phase of the clinical course (64).

**Figure 2:**
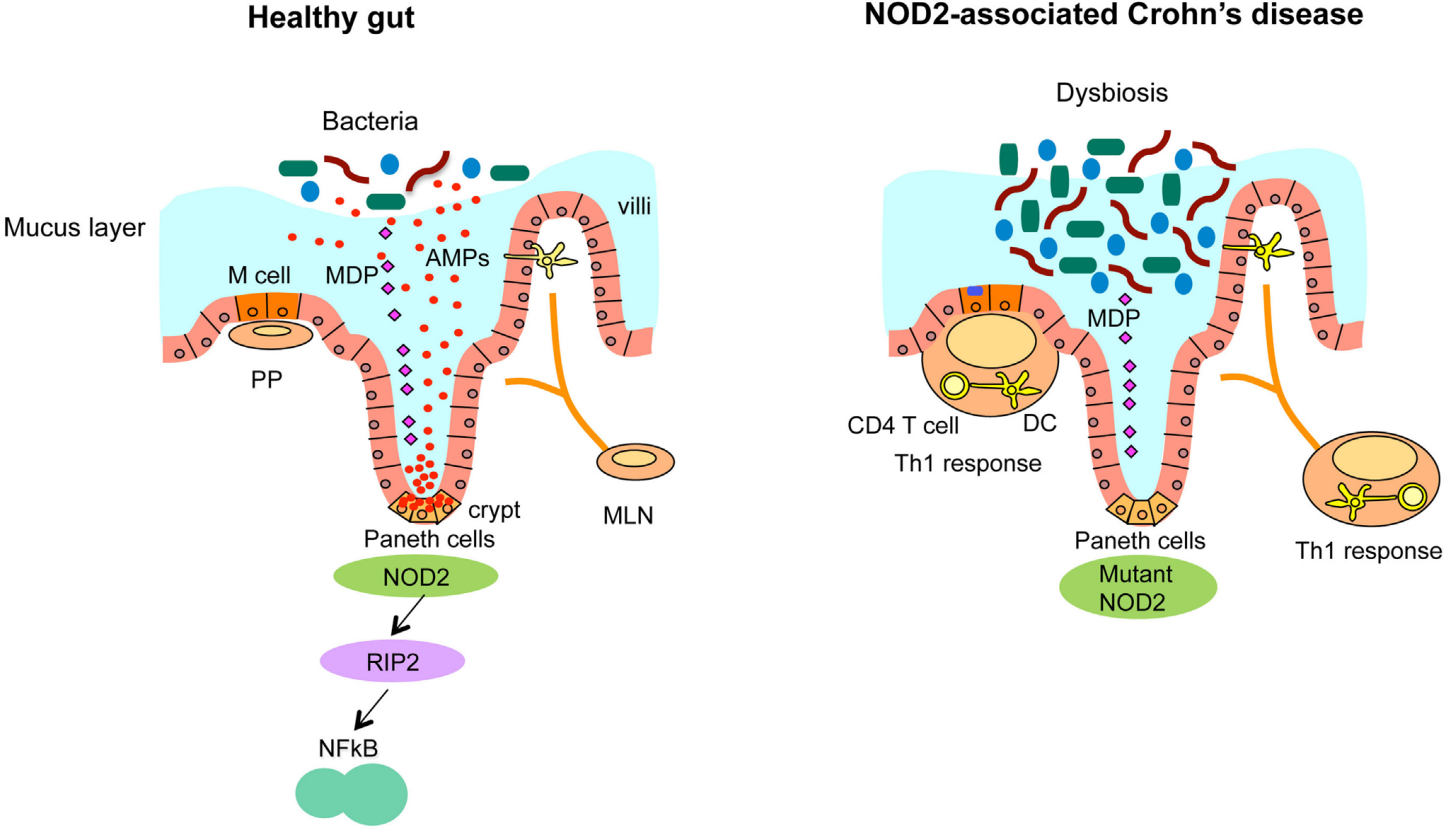
**NOD2-associated dysregulated microbiota leads to the susceptibility of CD**. Ileum in healthy individuals (left panel). NOD2 senses microbiota-derived MDP and activates NF-κB through the downstream kinase receptor-interacting protein 2 (RIP2), which is critical for the bacterial killing activity of Paneth cells via the secretion of anti-bacterial compounds. Ileum with loss of *NOD2* function (right panel). In CD, that is associated with *NOD2* mutations, CD-associated *NOD2* mutants disturb many characteristics of gut immune homeostasis, including reduced MDP sensing, and impaired antimicrobial responses in Paneth cells, leading to defective bacterial clearance. This dysbiosis caused by impaired Paneth cell function is characterized by increased load of bacteria and abnormalities of Peyer’s patches (PP) and mesenteric lymph nodes (MLN) that stimulate the mucosal immune system to induce Th1 immune response, leading to chronic inflammation.

In the healthy human intestine, the intestinal epithelium provides an effective barrier against luminal bacteria with the help of epithelial tight junction and the mucus layer (Figure 2). However, in patients with inflammatory bowel diseases, this barrier function is often compromised due to genetic, environmental, microbial, and immunological factors (65, 66). Dysfunctional mucosal barrier allows the penetration of bacterial products, leading to direct interaction with immune cells (Figure 2). Innate immune cells, such as macrophages and dendritic cells produce cytokines, such as TNF-α, IL-1β, IL-6, IL-12, IL-23, and chemokines upon recognition of microbes and microbial products. Activated dendritic cells promote the differentiation of CD4^+^ T cells into pro-inflammatory T cell subsets, such as Th1 or Th17 effector T cells, which secrete additional cytokines and chemokines (65, 66). Recently identified novel family of innate immune cells termed innate lymphoid cells (ILCs), in particular the type 1 ILC (ILC1) subset, produce high amount of pro-inflammatory cytokines, such as IFN-γ and TNF-α (67–69). High levels of chemokines produced by these innate and adaptive immune cells further recruit lymphocytes, creating a vicious positive feedback cycle of inflammation in CD.

## NOD2: The Highest Risk Factor in Ileal CD

Recent Genome-wide association studies (GWAS) revealed 163 susceptibility loci for IBD (inflammatory bowel disease), 30 of them being specific to CD (70). Among them, *NOD2* was the first gene identified as a risk factor for ileal CD (71, 72), discovered by the genetic mapping study of the CD susceptibility locus (73). One of the cell types that express *NOD2* at a high level is the Paneth cell, most of which are located in the terminal ileum (32). Paneth cells play an important role in the innate regulation of gut microbiota by synthesizing and secreting antimicrobial peptides or proteins. Upon the stimulation with bacterial products, such as the NOD2 ligand, MDP, Paneth cells secrete antimicrobial peptides, including lysozyme, secretory phospholipase A2 (sPLA2), and human α-defensins 5 and 6 (HD5 and HD6) in the intestinal lumen (74). Therefore, *NOD2* mutations lead to dysregulation of host–microbe interactions, which increases the susceptibility to abnormal ileal inflammation. In addition to this microbial dysbiosis, other genetic or environmental factors trigger the development of ileal CD.

A meta-analysis showed the risk for CD development was increased to 17.1-fold in *NOD2* homozygotes or compound heterozygotes, and 2.4-fold in simple *NOD2* heterozygotes (Table 1) (75). Three main variants or polymorphisms in *NOD2* gene (i) a frame shift mutation at position 1007 (1007fs); (ii) a glycine to arginine conversion at amino acid residue 908 (G908R); and (iii) an arginine to tryptophan conversion at amino acid residue 702 (R702W) were highly associated with susceptibility to CD (71, 72, 76). All three mutations are located within or close to the LRR domain that recognizes MDP. Indeed, patients with ileal CD showed reduced levels of HD5 and HD6 derived from Paneth cells (77, 78). Additionally, the production of α-defensins is particularly reduced in patients with non-functional *NOD2* mutations (79, 80). These findings showed that CD-related mutations in *NOD2* are loss of function and individuals carrying *NOD2* mutations are at high risk for ileal CD.

**Table 1:** ***NOD2* mutations in CD patients**.

Reference	*NOD2* mutation type	Risk for	Increase of risk
Economou et al. (75)	Single risk allele^a^	Development of CD	2.39-fold (OR = 2.39)
	Two or more risk alleles^a^	Development of CD	17.1-fold (OR = 17.1)
Cleynen et al. (81)	Any *Nod2* mutations	Ileal location	1.90-fold (OR = 1.90)
		Stricturing	1.82-fold (OR = 1.82)
		Fistulizing	1.25-fold (OR = 1.25)
		Need for surgery	2.96-fold (OR = 2.96)
Adler et al. (82)	Single risk allele^a^	Stricturing or fistulizing	8% (RR = 1.08)
	Two risk alleles^a^	Stricturing or fistulizing	41% (RR = 1.41)
	Any risk alleles^a^	Need for surgery	58% (RR = 1.58)
	G980R	Stricturing	33% (RR = 1.33)
Weersma et al. (83)	1007fs	Ileal location	1.83-fold (OR = 1.83)
		Stricturing	1.38-fold (OR = 1.38)
		Need for surgery	1.69-fold (OR = 1.69)
	G980R	Stricturing	1.65-fold (OR = 1.65)
Seiderer et al. (84)	1007fs	Early onset	73.7% (14/19 patients)
		Re-operation (re-stenosis)	78.6% (11/14 patients)

*^a^Risk allele: 1007fs, G908R or R702W*.

*OR, odds ratio; RR, relative risk*.

Furthermore, many studies have suggested that *NOD2* mutations increase the risk of complications in CD (Table 1). It was shown that any of the *NOD2* mutations are independent predictive factor for stricturing [odds ratio (OR) = 1.82], fistulizing (OR = 1.25), and need for surgery (OR = 2.96) (81). A meta-analysis reported that the risk for complicated disease, such as stricturing or fistulizing, was increased by 8% [relative risk (RR) = 1.08] in *NOD2* heterozygotes and 41% (RR = 1.41) in *NOD2* homozygotes or compound heterozygotes (82). Likewise, the risk of surgery was increased by 58% (RR = 1.58) with any of the *NOD2* mutations (82). In terms of each risk allele, p.1007fs mutation is the strongest disease predictive factor. p.1007fs was associated with stricturing (OR = 1.38) and the necessity of an operation (OR = 1.69) (83). The homozygous carriers of p.1007fs were significantly younger at the time of diagnosis (under the age of 26 years). Fourteen out of 19 homozygous carriers of p.1007fs (73.7%) required surgery and 11 out of 14 patients (78.6%) underwent the re-operation due to re-stenosis (84). The mutation of p.G980R is also associated with disease complications [RR = 1.33 (82), OR = 1.65 (83)]. Interestingly, multiple studies indicated that association of *NOD2* mutations with CD were found in European and Ashkenazi Jewish ancestry population, but not in CD patient cohort of Asian ancestry, such as Japanese, Chinese, Korean, or Indian, indicating the impact of ethnic genetic background on the CD susceptibility caused by *NOD2* mutations (85–88).

Taken together, *NOD2* mutation and the susceptibility of ileal CD are strongly related via altered interaction between ileal microbiota and mucosal immunity. Carrying the *NOD2* mutation is the strong risk factor for ileal CD with multiple complications.

## Role of Microbiota in Ileal CD

The surfaces of the body, particularly the gastrointestinal tract, are exposed to a large number of diverse microbes that are collectively referred to as the microbiota. Composition of the microbiota can be significantly affected by the genetic background of hosts along with several other factors, such as diet, age, stress, and diseases (89). While commensal bacteria colonize all mucosal surfaces and the skin, the intestinal tract harbors the largest bacterial load (up to 10^14^ total bacteria) (90, 91). It is becoming increasingly clear that the close symbiotic relationship between host and an adaptable microbiota forms a vital part of the intestinal homeostasis (92, 93). The host microbiota plays important roles in several processes such short-chain fatty acid production (94), the development of intestinal epithelium (95, 96), protection against invading pathogens, and, importantly, the development of mature immune system (96). The intestinal microbiota provides both inflammatory and anti-inflammatory products, which modulate the immune responses (97, 98) and dysbiosis in the microbiota is considered pivotal to the origin of many diseases. It is well known that the disease outcome can be influenced by the host microbiota either due to the increased colonization of pathogenic strains or the introduction of new bacterial species. This is well-exemplified in IBD patients who typically exhibit imbalances in the microbiota composition. Moreover, reducing exposure to intestinal bacteria in these patients has been shown to frequently alleviate inflammation (99, 100).

## Role of NOD2 in the Interaction Between Microbiota and Ileal Host Immunity

NOD2 plays an important role in mediating host–microbe interactions in the intestine. Interestingly, NOD2 has been shown to contribute to the homeostatic maintenance of the gut microbiota. Abnormal *NOD2* function associated with LRR polymorphisms leads to an inflammatory response mediated by innate immune functions (42, 71, 72, 101). NOD2 controls the expression and secretion of antimicrobial peptides thereby acting as an important regulator of the ileal commensal microbiota in mice (31). *Nod2-*deficient mice exhibit reduced bactericidal activity, higher loads of commensal bacteria and increased susceptibility to colonization by pathogenic bacteria (31, 102), and are, thus, prone to bacterial infections and intestinal inflammation (30, 42). NOD2 function, therefore, may be pivotal in understanding the balance between the host immune system and commensal bacteria.

## NOD2-Mediated Regulation of Ileal Microbiota

NOD2 is critical for regulating the bacterial flora in the ileum through the secretion of anti-bacterial compounds (31, 42, 103–105) (Figure 2). Earlier studies in *Nod2*-deficient mice indicated the role of NOD2 in the homeostasis of gut epithelium, possibly linking it to the development of CD (42). Furthermore, increased *NOD2* expression is observed at the sites for α-defensin secretion, such as intestinal crypts in mice (42) and Paneth cells in human (32, 33). *Nod2-*deficient mice harbor larger loads of bacteria in the ileum due to impaired Paneth cell function, compared to littermate controls even in the same cage (31). *Nod2-*deficient mice are susceptible to Th1-dominant granulomatous inflammation in the ileum, which is induced by an opportunistic pathogen *Helicobacter hepaticus* and ileal dysbiosis (2, 30). Furthermore, *NOD2* expression in the intestine is regulated by signals from the microbiota, given that germ-free mice had lower *NOD2* expression that was reversible upon monocolonization with commensal bacteria (31). Zhang et al. found that NOD2-mediated lysozyme trafficking in Paneth cells is directed by commensal bacteria, which is important for regulating intestinal infection (58). Two different steps, selective lysozyme trafficking and final secretion (106) are separately regulated by commensals implicating a new mechanism by which symbiotic bacteria direct a lysozyme-trafficking event to promote host defense against pathogens.

Several studies have shown the role of NOD2 in host protection against pathogens in a number of bacterial infectious models including *Listeria monocytogenes* (42), *Staphylococcus aureus* (107), *Chlamydophila pneumoniae* (108), *Streptococcus pneumoniae* (109), and *Mycobacterium tuberculosis* (110). *Nod2-*deficient mice were susceptible to *L. monocytogenes* infection via oral but not systemic routes, suggesting that NOD2 might have a non-superfluous role in intestinal antimicrobial responses (11).

The discovery that *NOD2* mutations are strongly associated with CD highlighted the importance of NOD2 in the regulation of antimicrobial responses. Loss of function mutations in *NOD2*, rendering it unresponsive to peptidoglycan component, MDP, have been implicated in the development of CD (41, 111, 112). Interestingly, patients with mutant variants of *NOD2* have altered microbiota in the ileum (32, 33, 113–117). There was a significant decrease in the relative frequencies of *Clostridia* and an increase in the *Actinobacteria* and *Proteobacteria* in CD patients compared to healthy controls (117).

Apart from having role in the bactericidal ability of Paneth cells, NOD2 also shapes the microbial community profiles via local production of reactive oxygen species (118–120). Adult *Nod2*-deficient mice display a substantially altered microbial community structure and a significantly elevated bacterial load in their feces and terminal ileum compared to their wild-type counterparts (114). Interestingly, these findings were also present in weaning-age mice, indicating a subtle influence of NOD2 on the early development and composition of the intestinal microbiota. Increased load of the *Bacteroidetes* and *Firmicutes* phyla as well as the genus *Bacteroides* was observed in the terminal ileum of CD patients with homozygosity in *NOD2* mutations (114). In addition, altered frequencies of *Faecalibacterium* and *Escherichia* have also been associated with *NOD2* risk alleles in CD patients. *Nod2-*deficient mice harbor increased bacterial load in the feces and ileum along with increased abundance of *Bacteroidetes* and *Firmicutes* in comparison to wild-type mice (31, 114, 121). These studies are supported by the observations of Petnicki-Ocwieja et al., who used the most strict experimental conditions taking into account the usage of littermates and bacterial quantification by qPCR, with the prominent difference observed only in ileum (31).

Several abnormalities in the small-intestinal epithelium of *Nod2-*deficient mice were investigated which included the inflammatory gene expression and goblet cell dysfunction (102). Ramanan et al. demonstrated that these abnormalities were associated with excessive interferon-γ production by intraepithelial lymphocytes (IELs) and Myd88-dependent signaling, and were dependent on the expansion of a common member of the intestinal microbiota, *Bacteroides vulgatus*. Although this study implies NOD2 prevents harmful immune responses by controlling the expansion of a pro-inflammatory member of the microbiota (102), the composition of the gut microbiota may vary among mice from different laboratories, which may lead to the variations in the experimental outcome (31, 114, 121).

## NOD2 and Gut Immune Homeostasis

The constant exposure of the intestinal tissue to gut microorganisms maintains the mucosa in a state of physiological inflammation, which balances tolerogenic and pro-inflammatory type responses to maintain homeostasis. In line with the idea that NOD2 signaling might indirectly regulate T cell populations, results presented in a recent study indicated that there are dysfunctional and reduced numbers of IELs in *Nod2-*deficient mice (122). These effects of *NOD2* deficiency could hamper the integrity of intestinal epithelium and lead to an altered immune response to the resident microbiota. A recent study showed that there is an increased sensitivity of *Nod2* as well as *Rip2*-deficient mice to DSS-induced colitis and colonic adenocarcinoma as a result of dysbiosis, and that the effect can be transmitted to wild-type mice through the microbiota (123). However, such differences in the composition of gut microbiota as observed in naive mice deficient in NOD2 signaling components is debatable as many other studies could not arrive at the same conclusion (124–126), depicting that, in accordance with the study in TLR-deficient mice (127), changes in the relative abundance of certain bacterial groups in *Nod2*-deficient mice emulate caging conditions and familial transmission rather than their NOD2 genotype. In regard to this discrepancy, the question of whether *NOD2* deficiency can cause different composition of intestinal bacteria requires additional study. Likewise, different reports in human studies suggest a reduced abundance of certain bacteria, including *Faecalibacterium prausnitzii* and other butyrate-producing organisms (128) in patients with colitis, but whether these changes occur before the onset of inflammation is an important question in the field. Continuing studies, such as these will help to determine whether dysbiosis occurs before disease onset and whether particular microbial profiles are indeed early biomarkers of disease susceptibility.

Studies conducted in healthy SPF mice support the observation in which failure of NOD2 to induce secretion of antimicrobial compounds allows for increase of certain bacterial groups. Studies by Petnicki-Ocwieja et al. (31) reported increased loads of *Bacteroides* and *Firmicutes* groups in the ileum of *Nod2-*deficient mice compared to wild-type/heterozygous mice from the same litter. However, no such difference was observed in the feces of these mice. This observation was attributed to the decreased bactericidal activity of crypt-secreted factors, such as α-defensins (42). Similarly, increased abundance of *Bacteroides* was also reported in the ileum of *Rip2-*deficient mice compared to wild-type littermates (31). However, Rehman et al. (114) reported immense abundance of *Bacteroidetes* in the feces of adult *Nod2-*deficient mice, whereas greater load of *Firmicutes* was observed only in the terminal ileum of these mice. Mondot et al. (121) reported increased colonic abundance of the *Rikenellaceae, Bacteroidaceae* and *Prevotellaceae* families in *Nod2-*deficient mice compared to wild-type mice with no specific housing conditions. In conclusion, these studies indicate the increased bacterial load in the ileum of *Nod2-*deficient mice although the variations observed in these studies can be attributed to different housing conditions.

Microbial dysbiosis in CD is likely to be shaped by the chronic inflammatory state of the intestine. Abnormal microbial composition in association with the detrimental genetic background increases the predisposition of *Nod2-*deficient mice to inflammatory diseases. Several studies on the microbiota in *Nod2-*deficient mice reveal its altered diversity as well as richness (114, 121). These studies emphasize the importance of NOD2 in regulating the intestinal microbiota composition and the subsequent outcome of CD.

Previous studies indicate an important role of NOD2 in the development of GALT (Gut associated lymphoid tissues) during the bacterial colonization of gut (129). In *Nod2-*deficient mice, high proportion of CD4^+^ T cells, increased inflammatory cytokine levels, and higher antigen and bacterial permeability rates (124) were observed, indicating an increased stimulation of Peyer’s patches (PP) by resident gut microflora. All these changes observed in these mice were suppressed after treatment with oral antibiotics. Therefore, apart from influencing the development of the GALT, NOD2 has a role in modulating the immune response by limiting the development of a Th1 immune response toward bacteria. The results described above support the observation that activation of NOD2 in dendritic cells controls their ability to induce a polarized Th1 response in CD4^+^ T cells (130), thus elucidating the role of NOD2 in hematopoietic cells. However, this cannot explain why the phenotype of *NOD2* mutation is only associated with ileal lesion. Also, the Th1 immune response phenotype was not observed in other studies unless colonized with *Helicobacter* species (30).

## Other Possible Mechanisms Caused by *NOD2* Mutations

Amendola et al. reported that the absence of spontaneous colonic inflammation in *Nod2-*deficient mice can be attributed to altered gut permeability caused by the high mucosal cytokine production and increased activity of MLCK. MLCK, myosin light chain kinase, is a factor that likely causes the increased gut permeability in *Nod2-*deficient mice due to its effects on tight junctions (131). *NOD2* deficiency, therefore, causes the permeability changes that increase the exposure of dendritic cells to factors, such as TLR ligands that in turn influence Treg cell development and subsequent changes in the microbiota. These results are in agreement with the studies in IBD patients where increased gut permeability and an altered microbiota are associated with *NOD2* polymorphisms with no inflammation (116, 117, 132, 133). Recently, it was found that *NOD2* is expressed in Lgr5+ stem cells using culture of intestinal crypt organoids, indicating that NOD2 may contribute to epithelial homeostasis by regulating stem cell function in addition to bacterial killing activity of Paneth cells (134).

Autophagy has emerged as a pivotal component of the innate immune response to intracellular bacteria and is also involved in the delivery of microbial ligands to intracellular compartments containing TLRs to trigger an antimicrobial response. Three groups revealed that autophagy is activated by NOD2 to augment intracellular bacterial killing (56, 57, 135). The studies by Travassos et al. (57) demonstrated that bacterial sensing by NOD proteins is linked to the induction of autophagy, thus providing a functional association between *NOD2* and *ATG16L1*, two of the most important genes associated with CD. Cooney et al. demonstrated that bacterial killing and MHC class II-dependent antigen presentation in primary human dendritic cells are increased by MDP-activated autophagy and this process involves ATG16L1, NOD2, and RIP2, but is independent of NLRP3 (56). Dendritic cells from CD patients expressing CD-associated *NOD2* or *ATG16L1* risk variants have the defect in autophagy induction, bacterial trafficking, and antigen presentation. Hence, *NOD2* polymorphism along with the defective lysosomal degradation, and impaired induction of antigen-specific CD4^+^ T cells would lead to bacterial persistence and thereby the inflammatory responses in CD (56). Furthermore, a recent study showed that ATG16L1- or NOD2-deficient DCs have impaired capability to induce Tregs upon stimulation with immunomodulatory molecules from the commensal *Bacteroides fragilis*, indicating the immunosuppressive role of NOD2 and ATG16L1 in intestinal dendritic cells (136). However, this suppressive function does not explain the critical role of NOD2 and ATG16L1 in Paneth cells and ileum specific inflammation (137).

*Nod2* deficiency in mice, thus, results in increased intestinal bacterial load and greater susceptibility to pathogenic bacterial colonization (31), which could result from either Paneth cell dysfunction, defects in intestinal autophagic responses, or more likely, a combination of the two. Thus, in addition to Paneth cell defects, *ATG16L1* and *NOD2* risk variants affect the intestinal epithelial cell antimicrobial responses, probably changing the gut microbiome and enhancing CD susceptibility (135).

## Perspectives

Although more studies are needed to fully understand the CD pathogenesis caused by *NOD2* mutations, recent studies have shown the important roles of NOD2 in the interactions between microbiota and the intestinal immune system. In the studies using *Nod2-*deficient mice, it was shown that NOD2 is critical for the function of bactericidal activity of ileal crypts and the regulation of ileal microbiota. Therefore, it is tempting to speculate that ileal CD caused by *NOD2* mutations is mainly due to the dysfunction of Paneth cells. Under physiological condition, microbiota in the ileum is tightly regulated by bacterial killing activity of Paneth cells in healthy individuals. This regulates both pathogenic and non-pathogenic bacteria as well as bacterial antigens that constitutively stimulate the intestinal immune system. An increased load of microbiota alone is likely not sufficient to induce CD pathogenesis, since inflammatory responses in mucosal immunity are tightly regulated by various mechanisms. However, in the presence of other genetic, environmental, and immunological factors, dysbiosis of bacteria may increase susceptibility to ileal inflammation by enhancing stimulation of the intestinal immune system beyond the controlled physiological threshold, leading to pathological changes and, finally, to chronic inflammation. Although the studies of Paneth cells and microbiota in *Nod2-*deficient mice and CD patients support this scenario, further research is required to fully elucidate the molecular mechanism of CD. Additional studies on CD-associated microbiota and animal models of ileal CD may provide a greater insight to develop better treatment and management methods of CD patients.

## Author Contributions

All authors listed have made substantial, direct, and intellectual contribution to the work, and approved it for publication.

## Conflict of Interest Statement

The authors declare that the research was conducted in the absence of any commercial or financial relationships that could be construed as a potential conflict of interest.
